# Serodiagnosis of human brucellosis by an indirect ELISA test using recombinant outer membrane protein 19 kDa (rOMP19) as an antigen

**DOI:** 10.1186/s12896-023-00817-2

**Published:** 2023-10-24

**Authors:** M. Golchin, S. Mollayi, E. Mohammadi, N. Eskandarzade

**Affiliations:** 1 Department of Pathobiology, School of Veterinary Medicine, Shahid Bahonar University of Kerman, Kerman, Iran; 2 School of Veterinary Medicine, Shahid Bahonar University of Kerman, Kerman, Iran; 3 Department of Basic Sciences, School of Veterinary Medicine, Shahid Bahonar University of Kerman, Kerman, 7616914111 Iran

**Keywords:** Brucellosis, Diagnosis, Indirect ELISA, Outer mnembrane protein 19 kDa

## Abstract

**Background:**

Brucellosis remains one of the global health concerns that reemerges in recent years. Delayed or inaccurate diagnosis end to a long treatment duration and financial burden; therefore, finding a good antigen for detection of specific anti-*Brucella* antibodies is crucial. We intended to evaluate the serodiagnosis value of recombinant *Brucella* outer membrane protein 19 kDa (rOMP19) using indirect ELISA system compared with Rose Bengal test.

**Results:**

The OMP19 sequence was successfully cloned into pET-28a and produced in *E. coli* cells (DE3). After extraction and purification of rOMP19, this protein was used for designing indirect ELISA to detect anti-*Brucella* antibodies in 73 human sera, including 6 brucellosis-positive and 67 brucellosis-negative samples. The accuracy of rOMP19 ELISA was evaluated by receiver operating characteristic (ROC) curve and then compared with Rose Bengal plate test and a commercial anti-IgG *Brucella* ELISA kit. In comparison with Rose Bengal plate test, the area under the ROC curve was 0.985 (95% CI, 0.96–1.00). From coordinates of the curve, the optimal cut-off value was selected at 0.147, in which the diagnostic sensitivity was 100%, and the specificity was 94%. At this cut-off point, 10 samples were diagnosed as positive (6 true positives and 4 false positives), while negative samples were all correctly diagnosed. The results of our designed rOMP19 ELISA was the same as data obtained from commercial ELISA kit, which applied LPS as an antigen.

**Conclusions:**

We concluded that OMP19 is an efficient antigen for the serodiagnosis of human brucellosis.

**Supplementary Information:**

The online version contains supplementary material available at 10.1186/s12896-023-00817-2.

## Background

*Brucella* is an intracellular pathogen that causes one of the most prevalent zoonotic infections, which is identified by undulant fever, arthritis, abortion, and testicular or bone abscess formation [1]. Individuals who consume contaminated dairy products or have unprotected contact with livestock, such as farmers/herdsmen, abattoir workers, laboratory staffs, and veterinarians are at high risk of brucellosis [2, 3]. Despite eradication efforts, the disease is still posing socioeconomic burdens for populations of livestock and people, especially in endemic areas [4]. According to Annual Epidemiological Report in 2020, brucellosis incidence rate varies from 0.03 to over 1000 cases per 100,000 population, respectively in countries where brucellosis was not previously prevalent and endemic regions [5]. The non-specific clinical symptoms and the low specificity of available diagnostic tests cause a high rate of misdiagnosis of brucellosis, which complicates the epidemiology of the disease; therefore, accurate diagnostic methods are still needed in this field [6, 7].

The bacterial culture is a gold standard test for confirmation of *Brucella*; however, in addition to a wide range of sensitivity, from 10 to 90%, this method is recognized as a high-risk method for laboratory personnel. Molecular biotechnology techniques such as real-time or AMOS-polymerase chain reaction (PCR) are designed to quantitatively detect small amounts of bacterial DNA or differentiate *Brucella* strains which are reliable if precise and expensive equipment is available [1]. Conventional approaches such as the Rose Bengal plate test (RBPT), Enzyme-Linked Immunosorbent Assay (ELISA), and the Standard-tube Agglutination Test (SAT) are the most popular methods that rely on using either whole bacterial antigen or LPS for detection of anti-*Brucella* antibodies [7]. The lipopolysaccharide (LPS) is a *Brucella* virulence factor, which is commonly used for ELISA development; however, cross-reactivity of antibodies against the *Brucella* LPS-O chain with other Gram-negative bacteria such as *Escherichia coli* O157, *Vibrio cholera*, *Francisella tularensis*, and *Yersinia enterocolitica* results in low specificity of this technique [8, 9].

Recently the advances in genetic engineering to substitute S-LPS resulted in the development of the novel ELISA tests based on the application of non-LPS *Brucella* immunodominant antigens either as a pure natural protein or multi-epitope recombinant proteins [8, 10–12]. Among them, *Brucella* outer membrane proteins with different molecular weight (groups I, II, and III with MW of 88–94, 36–38, and 25–34 kDa, respectively) have been recognized as potential candidates for application in brucellosis diagnosis techniques or vaccine design [8, 10, 12, 13]. For example, outer membrane protein 19 kDa (OMP19), which is conserved among most *Brucella* species, have been proven to be capable of conferring immunity in mice [12, 14]. In this regard, we attempted to design an indirect ELISA test for the detection of anti-*Brucella* antibodies using recombinant outer membrane protein 19 kDa (rOMP19) as an antigen.

## Results

### Preparation and characterization of rOMP19

**Fig. 1:**
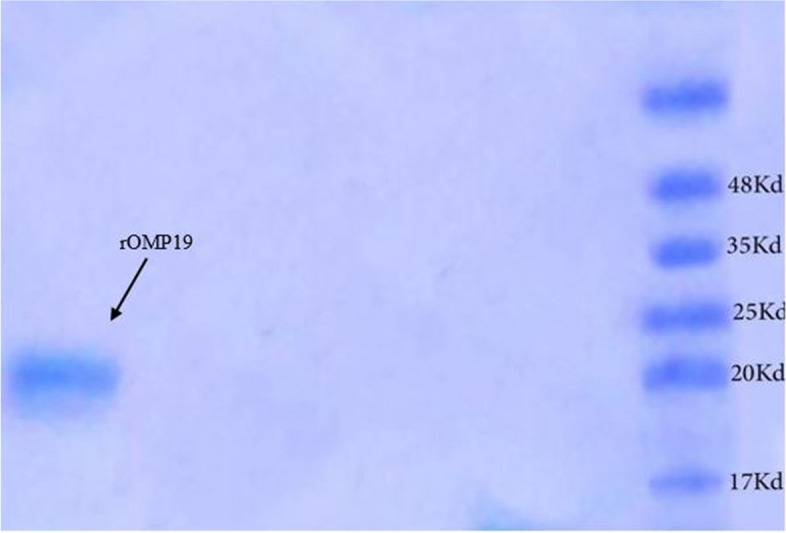
An expected protein product was specified by SDS-PAGE. Right lane: Protein prestained ladder. Left lane: Purified *Brucella* rOMP19 kDa

**Fig. 2:**
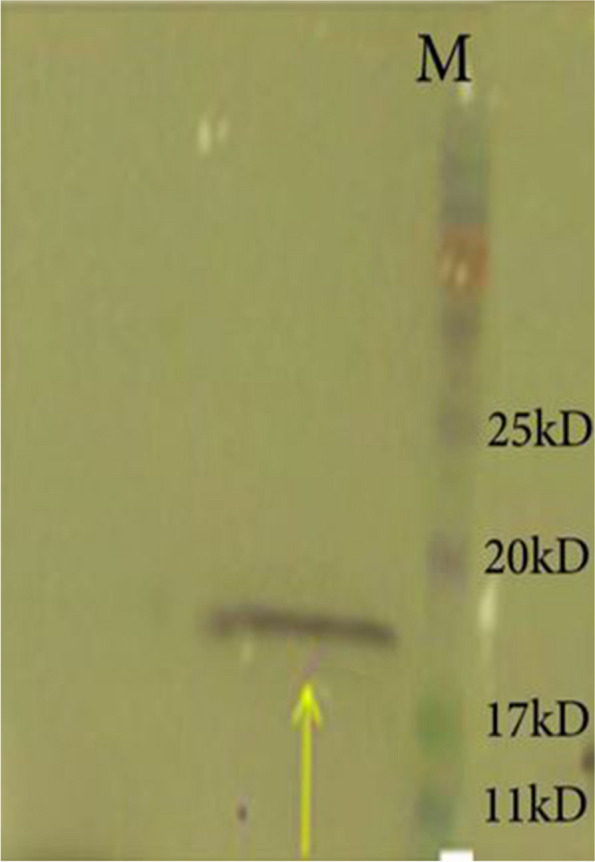
The rOMP19 protein was determined by commercial anti His-tag peroxidase-conjugated antibody in western blot. M: Protein pre-stained ladder. 1: Purified *Brucella* rOMP19 kDa

**Fig. 3:**
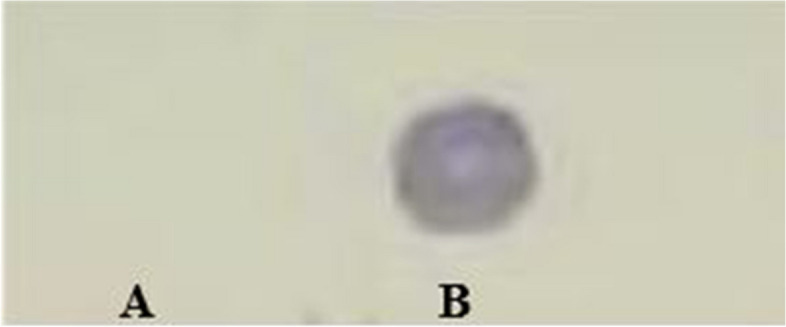
Purified His-tagged OMP19 protein was chased via dot blot technique using commercial anti-His-tag peroxidase-conjugated antibody. **A** indicates negative control (total cell lysate of *E. coli* that not prompted by IPTG) and **B**: shows rOMP19 protein

The cloning and expression of *B. abortus* OMP19 were achieved using vector pET-28a and prokaryotic expression system *E. coli*. The presence of expressed OMP19 was approved by a band close to the 20 kDa marker in a SDS-PAGE gel (Fig. 1). Western blot and dot blot analysis of soluble OMP19 preparations from *B. abortus* were shown in Figs. 2 and 3, respectively. The concentration of purified OMP19 was determined to be 0.8 mg/ml.

### Serological reactivity of rOMP19

**Fig. 4:**
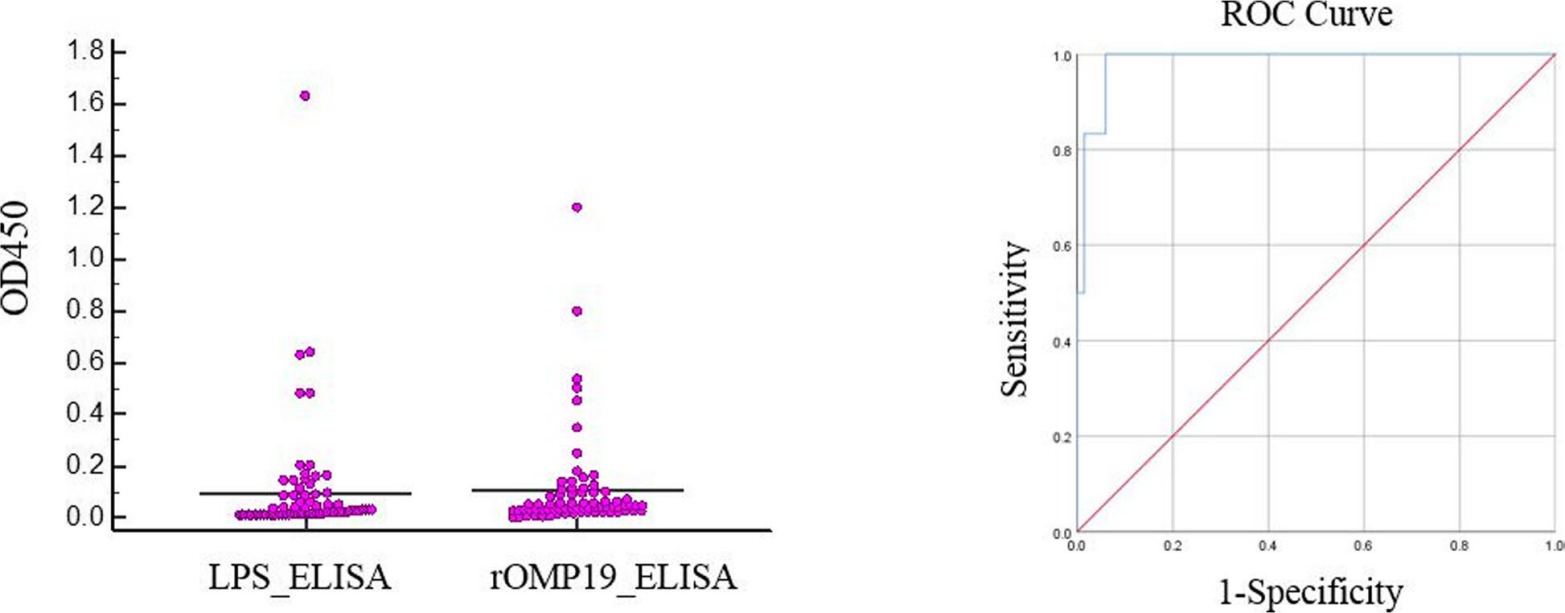
**A** Dot plot result of ELISA assays (LPS vs. rOMP19 antigen). **B** ROC analysis of rOMP19 ELISA compared with Rose Bengal test

A total of 73 brucellosis-suspected human sera, previously diagnosed as 6 positives and 67 negatives using the Rose Bengal plate test, were tested by rOMP19 ELISA. Reliability comparison of the rOMP19 ELISA with LPS ELISA and Rose Bengal plate test for *Brucella* antibodies detection is shown in Table 1. The AUC of ROC curve for OMP19 ELISA was 0.985 (95% CI, 0.96–1.00). Among multiple cut-off values suggested by Youden formula, the optimal cut-off value was selected at 0.147, in which the diagnostic sensitivity was 100%, and the specificity was 94% (Fig. 4). At this cut-off point, 10 samples were diagnosed as positive, while 63 samples were diagnosed as negative. Compared with the RBPT, the diagnostic accuracy of rOMP19 ELISA was 94% (69/73). The cut-off point of commercial LPS ELISA kit was estimated 0.158, based on the manufacturer's protocol. At this cut-off value, the diagnostic sensitivity of LPS ELISA kit was 100%, specificity was 94%, and accuracy was 94% (69/73) (Table 1).

**Table 1:** Reliability comparison of the rOMP19 ELISA and LPS ELISA with Rose Bengal plate test for *Brucella* antibodies detection

Cut-off value	Positive		Negative		Se%	Sp%
	TP	FN	TN	FP		
0.147^a^	6	0	63	4	100	94
0.158^b^	6	0	63	4	100	94

*TP* True positive, *TN* True negative, *FP* False positive, *FN* False negative, *Se* Sensitivity, *Sp* Specificity

^a^cut-off value is calculated for rOMP19 ELISA from ROC curve analysis

^b^cut-off value is calculated for LPS ELISA according to manufacturerʼs recommendation

## Discussion

Early and precise diagnosis of brucellosis via suitable antigen is a priority especially in endemic areas. In this study, we employed recombinant outer membrane protein 19 kDa (rOMP19) to develop an indirect ELISA test for detection of anti-*Brucella* antibodies in 73 human serum samples. Identification of the diagnostic accuracy of rOMP19 was performed by ROC curve, which showed AUC of 0.985. The optimal cut-off value was 0.147, in which the diagnostic sensitivity was 100%, and the specificity was 94%. At this cut-off value, there were 4 false positives but 0 false negatives. Compared with the accuracy of the RBPT, the diagnostic accuracy of OMP19 ELISA was 94%, which was the same as commercial LPS ELISA. Compared with the whole bacterial antigen used in Rose Bengal test, the rOMP19 had a strong sensitivity but a weaker specificity. Moreover, recombinant OMP19 showed strong negative predicted value (NPV = 100%), which means that the report of negative samples by OMP19 ELISA is valuable. High negative predictive value of a serological test is essential considering the fact that 31.8% of the clinically suspected cases, whom wrongly diagnosed as negatives, ultimately developed brucellosis [15].

Conversely, a low positive predictive value of serological tests can lead to the diagnosis of healthy cases as positive, which wrongly increases the total treatment cost [3, 16]. This problem can be addressed by screening for better cut-off value to prevent false results; for example, if we adjust the cut-off value of OMP19 ELISA to 0.297, sensitivity of the test decreases to 83% but the specificity increases to 98%, positive predictive value is also improved to 83%.

Another approach to improve the sensitivity and specificity of an ELISA test is the utilization of multi-epitope recombinant protein as antigen [17, 18]. For example, Yao et al. (2022) used OMP19 in different combination with *Brucella* outer membrane proteins in the serodiagnosis of brucellosis: (OMP10, OMP16, OMP19) and (OMP10, OMP16, OMP19, OMP25, OMP31 and BP26) [17]. They evaluated designed ELISA on 161 human sera and reported cut-off value 0.42 for both combination, and accuracy of 91.30% and 96.27%, respectively. These researchers suggested that the best OMP combination was omp25, omp31 and BP26 [17]. Dehghani et al. (2021) selected *Brucella* OMP22, OMP25, and OMP31 antigens and hybridized them with the rigid KP linker (K = Lysine, P = Proline) [18]. They evaluated their assembled antigen for diagnosis of brucellosis in 37 patients and 27 healthy individuals by ELISA tool. Using the ROC analysis, cut-off point, accuracy, sensitivity, and specificity for their recombinant protein were 0.809, 84.37%, 83.78%, and 88.89%, respectively [18]. Bulashev et al. (2020) produced recombinant OMP19 and studied immunogenic index of this protein in serum samples of mice immunized with rOMP19. They found that, a single injection of rOMP19 with no adjuvant, could induce the production of specific antibodies in mice after two weeks [12]. They also evaluated the antigenicity of rOMP19 using sera from 4 groups of animals: rabbits immunized with killed *B. abortus* 19 whole cell, experimentally infected cattle, vaccinated, and non-vaccinated cattle. They suggested that the ELISA designed to detect anti-OMP19 antibodies was more efficient than the ELISA tests developed for detection of anti-OMP25 or anti-OMP31 antibodies [12]. In the previous research conducted in our lab, we designed an indirect ELISA capable of detecting *Brucella* antibodies against OMP16. Out of 70 Brucellosis suspected human serum samples tested, eight samples were detected as positives and 62 as negatives at cut-off value of 0.13. Although the cut-off value of the rOMP16 ELISA (0.13) was lower than rOMP19 ELISA (0.147), accuracy of OMP16-based ELISA was 94%, which was close to 95% obtained in this study. Moreover compared to commercial ELISA, the accuracy was obtained 100% for both tests [8].

Simborio et al. (2015) determined the efficacy of combined recombinant *B. abortus* OMP10, OMP19 and OMP28 for serodiagnosis of bovine brucellosis by ELISA method [19]. The combined OMPs showed sensitivity, specificity and accuracy of 92.67%, 98.66%, and 96.04%, respectively. They concluded that assembled rOMPs were good candidates for diagnostic purposes, especially when it comes to differentiate *B. abortus* S19 vaccinated and naturally infected animals [19]. Because *Brucella* vaccine have not yet been developed for human, it seems that OMP19 could be applied for design iELISA test for human brucellosis.

## Conclusions

Due to the antimicrobial resistance crisis, early diagnosis of bacterial diseases deserves more attention. Brucellosis cases are on the rise globally; however, there is still a lack of accurate and specific diagnostic methods in this field. Laboratory methods apply S-LPS to trace *Brucella* antibodies, which cannot discriminate immunoglobulins in response to vaccine strains or some other Gram-negative bacterial infections. Accordingly, biotechnological approaches for production of *Brucella*-specific immunogenic proteins are necessary in this field. In this work, we showed that OMP19 could be an efficient antigen for developing the specific methods for the serodiagnosis of human brucellosis, albeit further experimental investigations are needed to confirm this result.

## Methods

### Expression and purification of the rOM19 protein

*Brucella* OMP19 gene sequence under Gene Bank accession number U35742 was synthesized (Generay Biotechnology, Shanghai, China) and cloned into pET-28a vector. The vector transformed into *E. coli* cells (DE3) by heat shock (42 ˚C for 90 s) and then *E. coli* cells harboring pET-28a plasmid was cultured (1:100) in a fresh LB medium with 100 μg/ml kanamycin (Sigma-Aldrich, USA) at 37 ˚C till the optical density at wavelength of 600 nm reached to 0.6 [8]. Recombinant protein expression using T7 RNA polymerase promoter was induced by adding 1 mM β-D-1-thiogalactopyranoside (IPTG, Pars Tous Co, Mashhad, Iran) for 20 h in a shaker incubator at 20 ˚C. Since the expressed OMP19 was secreted in the cytosol of bacteria, a lysis buffer containing sodium phosphate 50 mM, imidazole 10 mM, and NaCl 300 mM at pH = 8 followed by sonication (250 W, 10-s pulse on followed by 10-s pulse off, total time 4 min) were used for extraction. After centrifuge at 10,000 *g* for 45 min, an affinity chromatography by Ni–NTA Superflow pre-filled 1ml Cartridge (Qiagen, Hilden, Germany) was applied for purification of His-tagged protein from the supernatant. The native buffers for the nickel column were 50 mM Na phosphate; 300 mM NaCl; 10–400 mM imidazole, pH 8.0.

The concentration and characterization of the target protein was confirmed by different techniques including protein quantification at wavelength of 280 nm, electrophoresis on SDS-PAGE (12% acrylamide, according to Laemmli), blotting on nitrocellulose membranes, western blotting using anti-His Tag monoclonal antibodies conjugated with horseradish peroxidase (Sigma, St. Louis, USA). On the SDS-PAGE, we loaded 10 µl sample (diluted 1:4 with loading buffer) per well. Since the concentration of rOMP19 was 0.8 mg/ml, 1.6 µg protein was loaded per well.

### Serum sample collection

A total of 73 suspected human serum samples were collected in collaboration with a laboratory in Qaen County in south Khorasan province in Iran. These sera collected from human cases with fever of unknown origin and were tested by SAT method in the laboratory before beginning of this study.

We also used 20 negative serum samples (confirmed by RBPT, SAT, and anti-IgG *Brucella* ELISA kit) for technical assurance. All serum samples were tested with Rose Bengal test according to standard method. The RBPT antigen was purchased from Pasteur Institute, Iran. The suspension contained the inactivated *Brucella abortus* cells, which were stained with Rose Bengal. Briefly, the serum and RBPT antigen were mixed together in an equal volume on a plate and the results were read within 4 min. Medium to high degree of agglutination was considered as positive.

### Evaluation of sera with rOMP19 ELISA

Purified rOMP19 protein (100 µL, 50 µg/mL) was coated into the immunoassay plate (Nunc, Roskilde, Denmark) and then incubated at 4 ˚C, overnight. The wells were washed three times with PBS and then blocked with 200 µL of BSA 3% in PBS. PBS-diluted sera (100 µL, 1:100) were added to each well and were incubated for 1 h at 37 ˚C. After three washing steps with PBS-T (Merck KGaA, Darmstadt, Germany), the protein-coated wells were incubated with anti-Human IgG-HRP antibody (Serotec, Kidlington, UK) at dilution of 1:5000 for 1 h at 37 ˚C. In the next step, TMB (3,3',5,5'-Tetramethylbenzidine) substrate was added into the wells and the reaction was developed into the blue color if the anti-OMP19 antibodies were detected. Absorbance was read at wavelength of 450 nm by Anthos 2020 ELISA reader (Biochrom, Cambridge, UK). The background absorbance is also measured at 600 nm.

### Evaluation of sera with commercial ELISA

For evaluating *Brucella* LPS as an antigen for serodiagnosis of human brucellosis in comparison with rOMP19, all serum samples were evaluated with commercial *Brucella* IgG ELISA kit (Pishtazteb, Tehran, Iran) according to the manufacturer's instruction.

### Data analysis

Optical densities obtained from both ELISA tests were shown in a Dot plot using Medcal software. Receiver-Operating Characteristic (ROC) curve was obtained by SPSS software (version 26.9; IBM Corp., Armonk, USA). The area under the ROC curve (AUC) was collected to evaluate serodiagnosis efficacy of the rOMP19, and cut-off value was calculated using Youden index (specificity + sensitivity-1). Finally, true positives (TP), true negatives (TN), false-positives (FP), false negatives (FN), accuracy, positive predictive value (PPV) and negative predictive value (NPV) were estimated for both ELISA tests and compared with Rose Bengal test. The sensitivity and specificity of ELISA test were calculated using the following formulas:

$$\mathrm{Sensitivity}\:=\:\mathrm{Number}\;\mathrm{of}\;\mathrm{true}\;\mathrm{positives}/\mathrm{Number}\;\mathrm{of}\;\mathrm{true}\;\mathrm{positives}\:+\:\mathrm{number}\;\mathrm{of}\;\mathrm{false}\;\mathrm{negative}\;\mathrm{Specificity}\:=\:\mathrm{Number}\;\mathrm{of}\;\mathrm{true}\;\mathrm{negatives}/\mathrm{Number}\;\mathrm{of}\;\mathrm{true}\;\mathrm{negatives}\:+\:\mathrm{number}\;\mathrm{of}\;\mathrm{false}\;\mathrm{positives}$$

### Supplementary Information

**Additional file 1: Fig. 1.** An expected protein product was specified by SDS-PAGE. Lane 4: Protein prestained ladder. Lane 5: Purified *Brucella *rOMP19 kDa. **Fig. 2. **The rOMP19 protein was determined by commercial anti His-tag peroxidase-conjugated antibody in western blot. Left lane: Protein pre-stained ladder. Right lane: Purified *Brucella *rOMP19 kDa.

## Data Availability

Correspondence and requests for materials should be addressed to Mehdi Golchin.

## Abbreviations

PCR: Polymerase chain reaction
RBPT: Rose Bengal plate test
ELISA: Enzyme-Linked Immunosorbent Assay
SAT: Standard-tube Agglutination Test
LPS: Lipopolysaccharide
rOMP19: Recombinant outer membrane protein 19
TP: True Positives
TN: True Negatives
FP: False Positives
FN: False Negatives
PPV: Positive Predictive Value
NPV: Negative Predictive Value
ROC: Receiver-Operating Characteristic
AUC: Area under the curve
IPTG: β-D-1-Thiogalactopyranoside
