# Supplementary material for: Serodiagnosis of human brucellosis by an indirect ELISA test using recombinant outer membrane protein 19 kDa (rOMP19) as an antigen
Source: BMC Biotechnol. 2023 Oct 24;23:46. doi: 10.1186/s12896-023-00817-2 (PMC10598989; doi:10.1186/s12896-023-00817-2)
Supplement: Supplementary file 1 — Additional file 1: Fig. 1. An expected protein product was specified by SDS-PAGE. Lane 4: Protein prestained ladder. Lane 5: Purified Brucella rOMP19 kDa. Fig. 2. The rOMP19 protein was determined by commercial anti His-tag peroxidase-conjugated antibody in western blot. Left lane: Protein pre-stained ladder. Right lane: Purified Brucella rOMP19 kDa. [file 12896_2023_817_MOESM1_ESM.pdf]

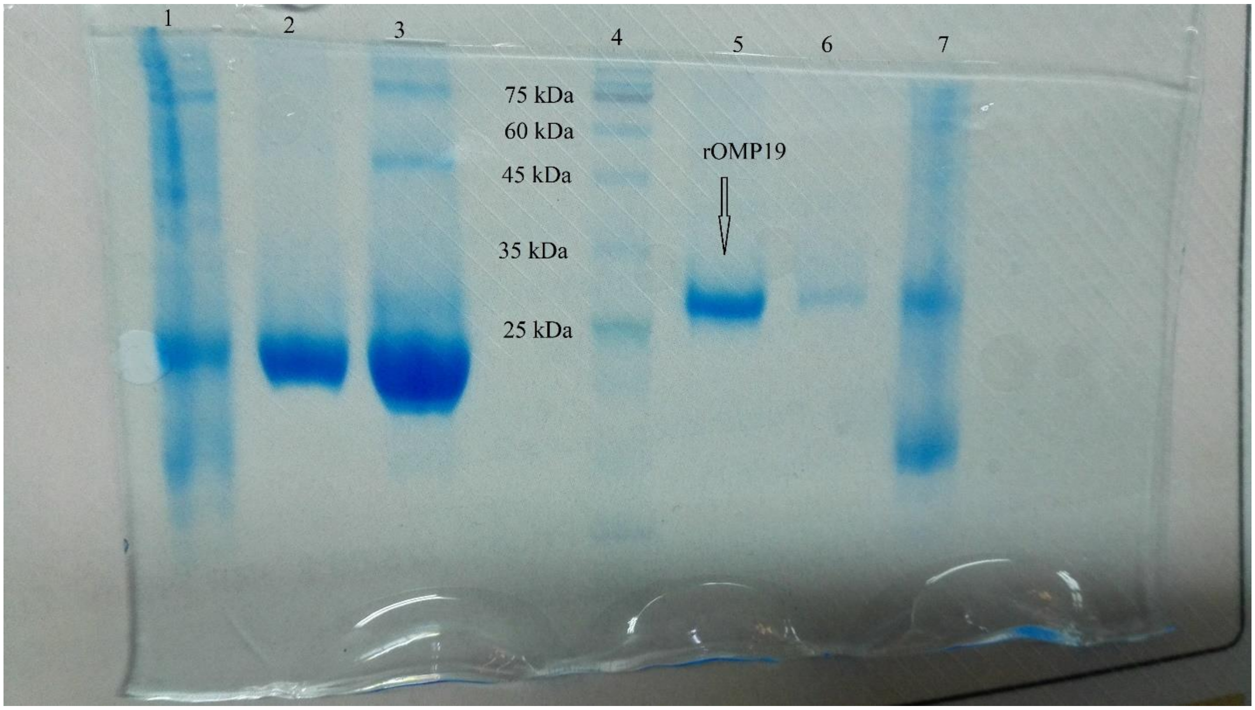

Fig. 1. An expected protein product was specified by SDS-PAGE. Lane 4: Protein prestained ladder. Lane 5: Purified *Brucella* rOMP19 kDa.

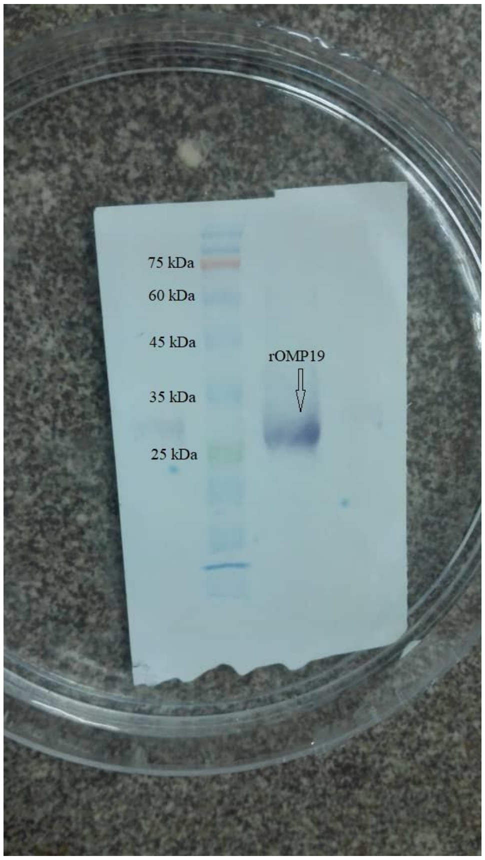

Fig. 2. The rOMP19 protein was determined by commercial anti His-tag peroxidase-conjugated antibody in western blot. Left lane: Protein pre-stained ladder. Right lane: Purified *Brucella* rOMP19 kDa.
